# Mr. Cullen Andrews Battle

**Published:** 1908-05

**Authors:** 


Mr. Cullen Andrews Battle, of Saint Louis, president of Battle & Company Chemists Corporation, died suddenly in his apartments at the Hotel Jefferson in that city, March 22, 1908, aged 59 years. Mr. Battle had been in indifferent health for some time, and he was on the eve of departure for a season of rest at his Michigan farm when death came. He had built up a successful business which maintained offices in Saint Louis, London and Paris. His brother and business associate, Mr. Jesse M. Battle, will undoubtedly become head of the house.

